# Rehabilomics Strategies Enabled by Cloud-Based Rehabilitation: Scoping Review

**DOI:** 10.2196/54790

**Published:** 2025-01-28

**Authors:** Sejun Oh, SangHeon Lee

**Affiliations:** 1 Korea University Anam Hospital Seoul Republic of Korea; 2 Human Behavior & Genetic Institute, Associate Research Center, Korea University Seoul Republic of Korea; 3 Department of Physical Medicine and Rehabilitation, Korea University Anam Hospital Seoul Republic of Korea

**Keywords:** cloud-based, health, rehabilitation, rehabilomics, strategies

## Abstract

**Background:**

Rehabilomics, or the integration of rehabilitation with genomics, proteomics, metabolomics, and other “-omics” fields, aims to promote personalized approaches to rehabilitation care. Cloud-based rehabilitation offers streamlined patient data management and sharing and could potentially play a significant role in advancing rehabilomics research. This study explored the current status and potential benefits of implementing rehabilomics strategies through cloud-based rehabilitation.

**Objective:**

This scoping review aimed to investigate the implementation of rehabilomics strategies through cloud-based rehabilitation and summarize the current state of knowledge within the research domain. This analysis aims to understand the impact of cloud platforms on the field of rehabilomics and provide insights into future research directions.

**Methods:**

In this scoping review, we systematically searched major academic databases, including CINAHL, Embase, Google Scholar, PubMed, MEDLINE, ScienceDirect, Scopus, and Web of Science to identify relevant studies and apply predefined inclusion criteria to select appropriate studies. Subsequently, we analyzed 28 selected papers to identify trends and insights regarding cloud-based rehabilitation and rehabilomics within this study’s landscape.

**Results:**

This study reports the various applications and outcomes of implementing rehabilomics strategies through cloud-based rehabilitation. In particular, a comprehensive analysis was conducted on 28 studies, including 16 (57%) focused on personalized rehabilitation and 12 (43%) on data security and privacy. The distribution of articles among the 28 studies based on specific keywords included 3 (11%) on the cloud, 4 (14%) on platforms, 4 (14%) on hospitals and rehabilitation centers, 5 (18%) on telehealth, 5 (18%) on home and community, and 7 (25%) on disease and disability. Cloud platforms offer new possibilities for data sharing and collaboration in rehabilomics research, underpinning a patient-centered approach and enhancing the development of personalized therapeutic strategies.

**Conclusions:**

This scoping review highlights the potential significance of cloud-based rehabilomics strategies in the field of rehabilitation. The use of cloud platforms is expected to strengthen patient-centered data management and collaboration, contributing to the advancement of innovative strategies and therapeutic developments in rehabilomics.

## Introduction

### Background

Rehabilitation is a crucial component of health care aimed at improving the functional capacity and quality of life for individuals facing various health challenges [1,2]. Traditionally, standardized approaches have been used in rehabilitation, sometimes failing to fully consider the unique needs and characteristics of each patient. However, the need for more personalized rehabilitation has become important after COVID-19 [3]. Advancements in genomics [4], epigenomics [5], proteomics [6], metabolomics [7], and related fields have made personalized rehabilitation approaches more feasible [8,9]. The convergence of rehabilitation and *omics* sciences has given rise to the concept of *rehabilomics* [10]. This concept uses an individual’s biological characteristics or biomarkers to tailor rehabilitation approaches, providing a pathway for personalized rehabilitation programs, and enabling the integration of “omics” for studying rehabilitation processes and outcomes [10,11].

Rehabilomics seeks to leverage cutting-edge molecular and genomic information to tailor rehabilitation interventions for optimal patient outcomes [10,12]. By understanding the genetic, proteomic, and metabolic profiles of patients, health care providers can design rehabilitation programs that are not only more effective, but also better aligned with an individual’s genetic and physiological makeup [11-13]. This paradigm shift holds immense promise for improving rehabilitation outcomes and enhancing patient experiences [14].

Rehabilomics can build systematic patient information based on large data using cloud-based solutions, use data from medical experts through patient consent, and establish customized treatment and rehabilitation strategies for patients [10,15]. In addition, evaluation- and monitoring-based rehabilitation interventions can be performed at home by collecting quantitative and objective information [16,17]. Owing to the nature of the cloud, there are advantages, such as simultaneous data sharing through the network; however, if the security measures are insufficient, there may be problems, such as data loss or patient information leakage; therefore, it is necessary to prepare for establishing data management system functions and security [18].

Simultaneously, the proliferation of cloud computing technologies has revolutionized data storage, sharing, and analysis in the health care domain based on the Fourth Industrial Revolution [19,20]. Cloud-based solutions offer scalable, secure, and cost-effective platforms for managing vast amounts of patient data, including genomic and clinical information [21]. Particularly, blockchain-based cloud solutions ensure a user-centric electronic health record system, offering enhanced security controls and a platform that securely safeguards health care data, emphasizing privacy and stability [22,23]. This technological convergence of rehabilomics and cloud computing opens up new horizons for data-driven rehabilitation practices, facilitating data sharing among health care professionals (rehabilitation medicine specialists, physical therapists, occupational therapists, speech-language pathologists, and other advocates of rehabilitation-related professionals), researchers, and patients [24].

Despite these exciting possibilities, there remains a need to comprehensively explore the current landscape of cloud-based rehabilitation in the context of rehabilomics [25].

### This Review

This scoping review aims to fill this gap by providing an overview of the existing literature and shedding light on key trends, challenges, and opportunities in this emerging field. By examining the integration of cloud-based technologies with rehabilomics strategies, we aim to contribute to understanding how these approaches can be harnessed to optimize rehabilitation interventions and ultimately improve patient outcomes. Therefore, this study examined the latest trends through a scoping review of cloud-based rehabilitation. Accordingly, we sought to derive strategies for rehabilomics. The rehabilomics approach, which uses an individual’s genomic, proteomic, and metabolomic information to deliver personalized rehabilitation, has tremendous potential to enhance the effectiveness and patient experience of rehabilitation services. Moreover, the integration of cloud computing technology can facilitate data sharing and collaboration among health care providers, researchers, and patients, thereby opening opportunities to develop innovative, data-driven rehabilitation solutions.

## Methods

### Literature Search Strategy

The primary objective of this scoping review was to investigate and synthesize current literature trends in cloud-based rehabilitation for rehabilomics strategies. To initiate the research, we conducted searches across major academic databases, including CINAHL, Embase, Google Scholar, PubMed, MEDLINE, ScienceDirect, Scopus, and Web of Science. The search terms and syntax were designed to encompass terms such as “cloud-based rehabilitation,” “rehabilomics,” “cloud computing in rehabilitation,” “personalized rehabilitation,” and “omics technologies in rehabilitation.” The search included articles that were published between January 2000 and July 2024. The literature review was conducted using a 2-step process. First, we examined the abstracts as a preliminary step toward gaining an initial understanding. This was followed by a more detailed review of the full-text of the selected articles, based on insights gathered from screening the abstract. The study protocol was registered in the Open Science Framework [26,27]. The research questions were as follows:

- Have cloud-based rehabilitation systems been developed and evaluated recently?
- What rehabilomics strategies are based on primary outcomes and user perceptions of cloud-based rehabilitation systems?

### Inclusion and Exclusion Criteria

The study adhered to established reporting guidelines (Multimedia Appendix 1), including the PRISMA (Preferred Reporting Items for Systematic Reviews and Meta-Analyses) Extension for Scoping Reviews (PRISMA-ScR). Scoping reviews aim to summarize the breadth of evidence on a topic and identify gaps in future studies. Consistent with best practices for scoping reviews, the authors designed and registered a priori protocol in the Open Science Framework, ensuring rigor and transparency throughout the review process.

Inclusion and exclusion criteria were established to select articles from the search results. The inclusion criteria included papers discussing the integration of cloud computing and rehabilomics strategies, applications in the fields of rehabilitation and health care, data sharing and collaboration, and personalized therapy. Papers unrelated to relevant topics or those lacking sufficient information were excluded. Two independent reviewers conducted a thorough examination of the papers, and the final selections were made through discussion.

Studies were only included when (1) full texts were available and (2) cloud-based rehabilitation was used. Owing to the broad nature of this scoping review, we were not limited by participants, study design, intervention, or outcomes. Exclusion criteria were as follows: (1) full text was not available, (2) papers which were not in English, (3) cloud-based rehabilitation was not used in the study, (4) unclear rehabilomics, and (5) studies focusing only on rehabilitation.

### Data Extraction and Analysis

Relevant information was extracted and analyzed from the selected papers. This includes details such as paper titles, authors, publication years, research topics, methodologies, key findings, and conclusions. Extracted data were synthesized in relation to key themes and subjected to statistical and content analyses to ascertain current research trends in cloud-based rehabilitation and rehabilomics strategies. The data were analyzed by 2 researchers, of whom one had >15 years of experience as a physical therapist and 20 years of experience as a rehabilitation medicine specialist. The data were analyzed through a basic literature search and review, a detailed review and screening, and a final review and decision.

### Reporting of Findings

The results from this scoping review are presented as a comprehensive overview of key themes, research trends, and related topics. This review summarizes the current research trends in cloud-based rehabilitation and rehabilomics strategies and offers suggestions for future research. Overall, 214,040 databases were searched, 2915 records were screened, 58 were retrieved, and 31 were assessed for eligibility.

## Results

### Characteristics of Included Studies

**Figure 1:**
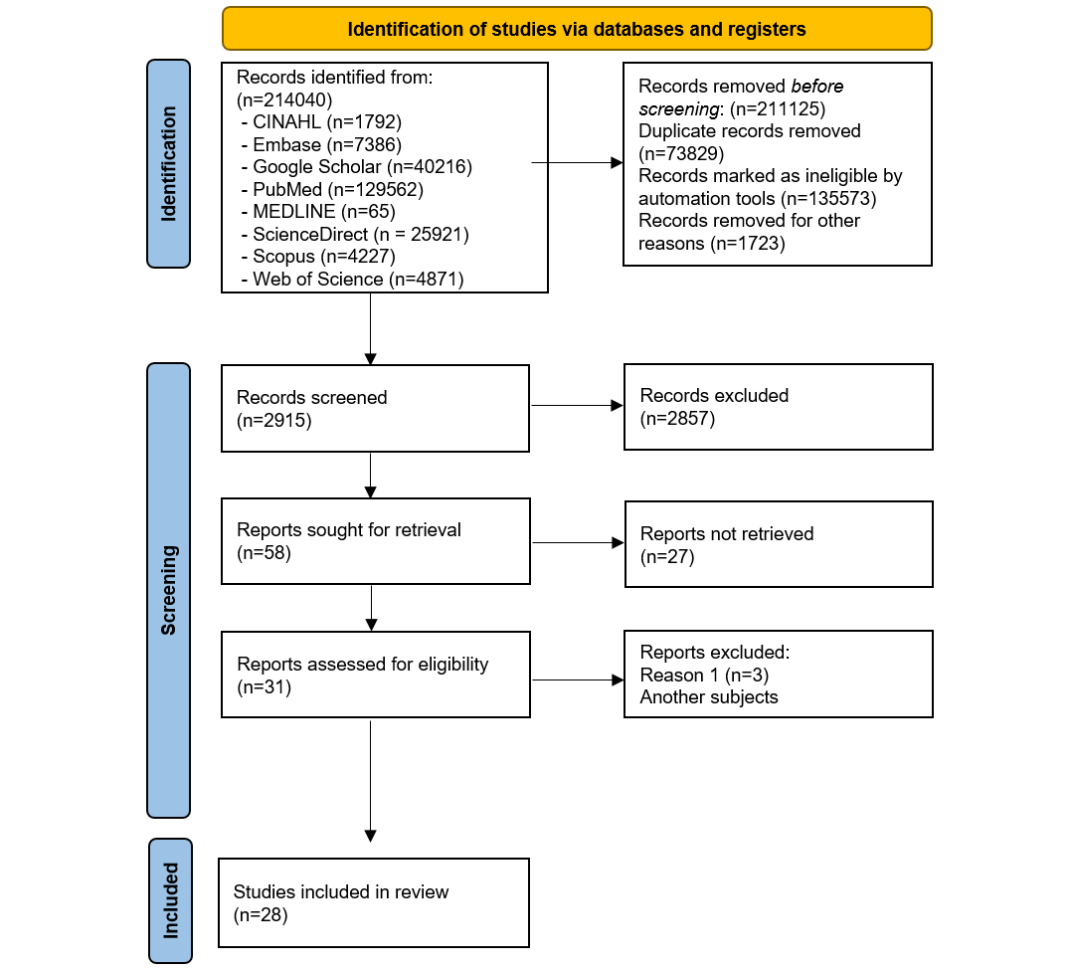
PRISMA (Preferred Reporting Items for Systematic Reviews and Meta-Analyses) diagram to identify studies using databases and registers.

These studies cover a wide range of topics related to cloud-based rehabilitation and rehabilomics strategies (Multimedia Appendix 2). Figure 1 provides an overview of the selection process based on the PRISMA diagram to identify studies using databases and registers.

### Key Themes

#### The Analysis of the Included Studies Revealed Several Key Themes

##### Integration of Cloud Computing

A total of 28 of the included studies [16,17,28-53] explored the integration of cloud computing technologies in the context of rehabilitation and rehabilomics. Cloud platforms are used for data storage, analysis, and sharing to facilitate collaborative research efforts.

##### Personalized Rehabilitation

Out of 28 studies, 16 (57%) studies [16,17,28,29,33-35,38,39,42,46,48,49,52,53] focused on the concept of personalized rehabilitation by leveraging omics data to tailor rehabilitation programs for individual patients. This approach aims to improve treatment efficacy and patient outcomes.

##### Data Security and Privacy

Out of 28 studies, 12 (43%) studies [16,17,28,34-36,41,44,47,49,52,53] addressed the critical issues of data security and patient privacy in cloud-based rehabilitation. Ensuring the confidentiality and integrity of patient data has become a key concern.

##### Interdisciplinary Collaboration

Collaboration between researchers from diverse backgrounds, including rehabilitation specialists, geneticists, and data scientists, was a recurring theme [16,17,28-53]. Such interdisciplinary collaboration is essential for advancing this field.

### Cloud-Based Rehabilitation

#### Overview

**Figure 2:**
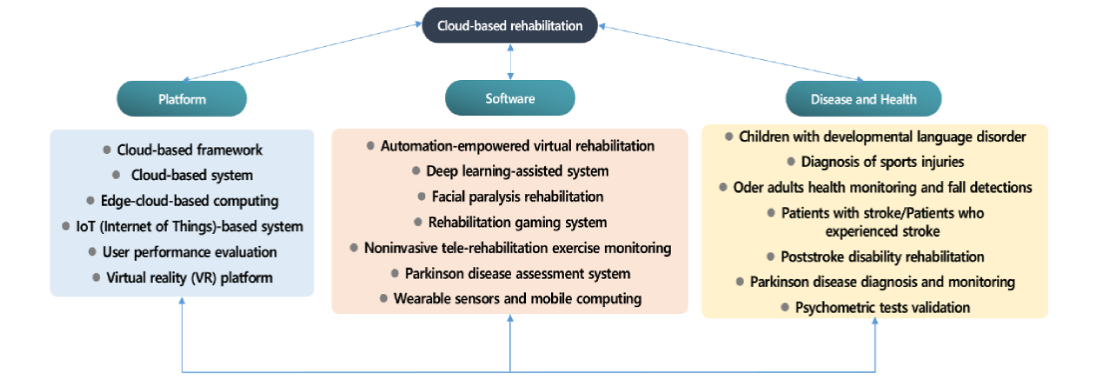
Trends of cloud-based rehabilitation.

We identified and included a total of 28 studies focused on cloud-based rehabilitation. These studies can be categorized into platform, software, and disease and health topics (Figure 2).

Because of the synthesis of the contents of the categories of these studies, the platform included cloud-based frameworks [28], cloud-based systems [2,3,14,23,29], Internet of Things (IoT)–based systems [12,30], user performance evaluation, virtual reality (VR) platforms [19,31], and edge-cloud–based computing. Software included rehabilitation gaming systems [20,22], noninvasive telerehabilitation exercise monitoring [31], Parkinson disease (PD) assessment systems [6], wearable sensors [10], mobile computing [5], deep learning-assisted systems [16], facial paralysis rehabilitation [25], and automation-empowered virtual rehabilitation [33]. Diseases and health conditions included stroke, poststroke disability rehabilitation [8,21,24], neurorehabilitation [13], PD diagnosis and monitoring [17], psychometric test validation [47], developmental language disorders in children [51], and diagnosis of sports injuries [52]. It also included health monitoring and fall detection in older people [35].

#### Cloud

##### CRehab

This refers to a cloud-based framework for rehabilitation management [28].

The authors introduced “CRehab,” a cloud-based framework featuring web-based and augmented reality support aimed at managing the rehabilitation processes. This framework targets the administrative, communication, and patient-motivation aspects by leveraging cloud capabilities. The primary objective was to devise an architecture adaptable to diverse rehabilitation types, irrespective of the diagnosis, environment, or patient demographics.

##### Rehab-aaService

This refers to a cloud-based motor rehabilitation digital assistant [29]. The integration of body sensor networks with cloud computing for motor rehabilitation enables remote monitoring, reduces costs, and provides comfort and safety to patients. Rehab-aaService, the newly introduced motor rehabilitation digital assistant, is based on wearable motion sensor nodes, a personal mobile device, and a cloud-based backend, offering enhanced support and a more comprehensive rehabilitation experience.

##### Open-Source Cloud-Based Platform for Health Monitoring and Fall Detection in Older People

The authors designed an open-source eHealth monitoring and fall detection system that integrated medical sensors, a microcontroller, and cloud communication [35]. This system stores data in the cloud to aid diagnosis, real-time monitoring, and rehabilitation facilitation. In addition, it features a fall detection system that promptly alerts medical personnel in the event of unexpected falls.

#### Platforms

##### Cloud-Based Rehabilitation Exergames System

A low-cost rehabilitation gaming system using Microsoft Kinect was developed for individuals with chronic arm pain [30]. This system encompasses goal-oriented exercises, enabling accurate performance measures for quantitative evaluation of the treatment plan. It notably boosts user motivation, hastens muscle recovery, and provides therapists with a means to assess patient progress.

##### Development of a VR Platform for Cloud-Based Neurorehabilitation

The authors designed a cloud-based VR platform for neurorehabilitation to analyze its impact on the sense of agency and ownership in healthy individuals [40]. The experimental platform featured a virtual avatar that replicated the individual’s movements using motion capture technology and a head-mounted display. The findings revealed that perceived arm length changed according to the displayed arm length of the avatar, demonstrating the considerable potential of the VR system.

##### SIGVERSE: a Cloud-Based VR Platform for Human-Robot Interaction Research

The authors introduced a cloud-based VR platform to explore human-robot interaction and obtain social intelligence for robots [50]. This successfully showcased the system’s practicality in real human-robot interaction scenarios, indicating its potential for fostering social intelligence development.

##### Edge-Cloud–Based Wearable Computing for Automation-Empowered Virtual Rehabilitation

This study referred to technology-enhanced rehabilitative care for patients with stroke through the use of edge cloud and IoT technology [53]. This involved the development of an edge-cloud model to track the vital signs and rehabilitation progress of patients in real time. The system exhibited remarkable operational efficiency and significantly improved rehabilitation treatment outcomes, paving the way for more advanced and intelligent medical service systems.

#### Hospitals and Rehabilitation Centers

##### Cloud-Based Rehabilitation System for Patients With Stroke

A cloud-based rehabilitation system focused on improving motor function in patients with stroke was developed. This study used data from healthy participants to accurately assess hand function [33]. Using the autoregressive integrated moving average model with the dynamic time-warping algorithm, this study made predictions regarding the rehabilitation and recovery of patients with stroke. Over a 10-week period involving 3 patients, the system showed significant improvements in upper limb control.

##### Monitoring of Upper Limb Rehabilitation and Recovery after Stroke: An Architecture for a Cloud-Based Therapy Platform

The Limbs Alive project was introduced at the Newcastle University and is dedicated to using video games for therapeutic movements to assist patients with stroke [34]. The authors developed a cloud-based therapy platform that gathers data from therapeutic games played by patients with stroke, estimates performance metrics, and delivers results to patients and clinicians via web applications.

##### System-Level Design of a Cloud-Based Training Device for Upper Limb Spasticity Rehabilitation

A robotic part-task trainer was developed to evaluate upper limb spasticity using cloud-stored clinical data [39]. It enables the remote coaching of trainees and continuous updates by rehabilitation physicians, thereby fostering a dynamic learning environment.

##### Cloud-Based Training and Evaluation System for Facial Paralysis Rehabilitation

This study referred to focusing on aiding facial paralysis rehabilitation via cloud-based information and communication technologies [46]. The proposed system aims to support patients and physicians by delivering rehabilitation training, automating progress reviews, and evaluating the results. It involves training a client for patient rehabilitation and data collection, leveraging machine learning on a cloud platform to analyze outcomes.

#### Telehealth

##### Cloud-Based Noninvasive Telerehabilitation Exercise Monitoring

The authors developed a conceptualized cloud-based telerehabilitation framework using cost-effective, noninvasive Microsoft Kinect for rehabilitation exercises [31]. This framework enables patients to engage in rehabilitation exercises at home while maintaining high-quality care standards. The initial experiments validated the potential of the system for training healthy participants to mimic physical rehabilitation exercise motions.

##### Mobile Cloud–Based PD Assessment System

The authors designed “PD Dr,” a mobile health app aimed at gathering PD-related motion data and evaluating symptoms [16]. The system was examined using information from 40 patients with PD to validate its efficiency in capturing significant symptoms and estimate their severity. It demonstrated robust connections between the collected motion features and the severity of PD, hand-resting tremors, and gait difficulty.

##### Cloud-Based PD Diagnosis and Monitoring Framework

The authors suggested a cloud-based framework designed to detect and monitor patients with PD, particularly in low-resource settings [17]. The goal was to provide remote diagnosis for patients in areas with limited health care access, achieving high accuracy in the process.

##### Remote Cloud-Based Automated Stroke Rehabilitation Assessment Using Wearables

These studies showcase a broad spectrum of cloud-based rehabilitation applications, ranging from pediatric developmental language disorder rehabilitation to stroke rehabilitation assessments using wearable technology [45]. These efforts aim to enhance rehabilitation outcomes through innovative technological approaches and data-driven analyses by incorporating cloud computation and sensor-based data for accurate assessment.

##### IoT Cloud-Based Telerehabilitation Service for Smart Cities

This study was centered on telerehabilitation as a service by leveraging cloud computing, IoT, and big data analytics [49]. It emphasizes the management of significant health care data generated from remote rehabilitation devices within the hospital cloud using NoSQL databases.

#### Home and Community

##### Integration of Wireless Wearable Sensors and Mobile Computing with Cloud-Based Service for Patient Rehabilitation Monitoring

The authors proposed a system that used wearable wireless sensors and handheld devices to track body motion, focusing on patients with stroke [36]. This setup allowed patients to conduct rehabilitation exercises at home while health care providers remotely monitored their progress. This system integrates cloud services for remote monitoring, eliminating the need for expensive hardware and software licenses for data storage. Initial user tests demonstrate the feasibility of the system.

##### IoT-based System for Physical Rehabilitation Monitoring

The authors described an IoT-based wearable system designed for monitoring physical rehabilitation using accelerometer and gyroscope sensors [37]. This system can record movement data to characterize patient movement and estimate their condition, thereby enabling remote monitoring through a cloud-based service with accurate capabilities for monitoring elbow rehabilitation characteristics.

##### Cloud-Based Platform for Autonomous Home Rehabilitation With Exergames

The authors developed a cloud-based platform that facilitates independent at-home rehabilitation through customizable exercise games [43]. This system uses a flexible database and structured rehabilitation data to allow clinicians to review and fine-tune exercise parameters efficiently. The platform incorporates parameterized exercise games designed to offer personalized goal-oriented rehabilitation.

##### Cloud-Based Smart Home Environment for Home Health Care

The authors developed “CoSHE,” a cloud-based smart home environment for monitoring the health of older populations within their homes [44]. The system incorporates wearable sensors and environmental data to provide real-time information to remote caregivers. The integration of contextual information successfully enhances understanding of an individual’s health status.

##### User Performance Evaluation and Real-Time Guidance in Cloud-Based Physical Therapy Monitoring and Guidance System

A cloud-based physical therapy monitoring and guidance system was developed to address the limitations of traditional therapy owing to the restricted interaction time [48]. The system leverages the actions of a physical therapist recorded as an avatar to guide patients in real time for self-training. By aligning the physical therapy and user motion sequences with a gesture-based dynamic time-warping algorithm, it evaluates user performance based on the physical therapy criteria and provides real-time guidance, optimizing patient learning.

#### Disease and Disability

##### Speech-Controlled Cloud-Based Wheelchair Platform for Disabled Persons

A prototype speech-controlled cloud-based wheelchair platform using WebKit Speech Application Programming Interface in the cloud was developed, which introduced a graphical user interface compatible with the web and mobile devices that enabled live video streaming [32]. It achieved speech recognition accuracy between 60% and 97% and recorded latencies spanning from 0.4 to 1.3 seconds. Through clinical testing, it exhibited promising outcomes, signaling potential for future implementation.

##### Cloud-Based Architecture Proposal for Aphasia Rehabilitation

The authors designed a cloud-based architecture specifically tailored for aphasia rehabilitation in Romanian-speaking patients [38]. The system comprises an application database, logic, and interfaces for patients and therapists. Using a virtual assistant, the system guided patients through exercises crafted by therapists. Treatment adjustments were made based on the obtained scores and the data were stored in a statistical module for further assessment.

##### Comparison of Python and Java Capabilities for Somatosensory Games in Cloud-Based Rehabilitation

The authors investigated the performance and stability of cloud-based somatosensory games in rehabilitation systems, while handling various user loads [41]. In this study, Python and Java programming languages were compared, and the findings indicated that Python consumed fewer resources in both the simulation and monitoring systems.

##### Cloud-Supported Framework for Poststroke Disability Rehabilitation

The authors suggested a cloud-based rehabilitation framework that combined augmented reality and sensor gloves for patients after an episode of stroke [42]. Notably, considerable enhancements in finger strength were observed after 6 weeks of therapeutic exercise. Sensor gloves were used to recognize the real-time conditions of the patients engaging in rehabilitative exercises.

##### Cloud-Based Flexible Solution for Psychometric Test Validation, Administration, and Evaluation

A unified cloud-based resource was developed to manage psychometric tests used for attitude evaluation, personal selection, educational purposes, rehabilitation, and diagnosis of cognitive disorders [47]. This resource aims to simplify the process of validation, standardization, and reorganization of tests traditionally conducted using complex statistical methods.

##### Effectiveness of Cloud-Based Rehabilitation in Children With Developmental Language Disorders During the COVID-19 Pandemic

This study referred to the application of cloud-based rehabilitation for children with developmental language disorders during the COVID-19 pandemic [51]. The authors investigated the effectiveness of the JingYun Rehab Cloud Platform on language and cognitive outcomes in 162 children with developmental language disorders through a prospective cohort study. Patients who underwent remote cloud-based rehabilitation were compared with a control group that received home-based rehabilitation. The results indicated significantly better language abilities among the children in the cloud-based rehabilitation group.

##### Cloud-Based Deep Learning–Assisted System for Diagnosis of Sports Injuries

The authors proposed a deep learning-assisted system incorporating IoT sensors in a body area network to diagnose sports injuries using cloud computing resources. IoT sensors gather data for diagnosis of injury and cloud computing to provide flexible computing resources [52]. They developed a brain injury monitoring framework and optimized neural networks to forecast sports injuries and evaluated the model performance using accuracy, precision, recall, and *F*_1_-score metrics.

**Figure 3:**
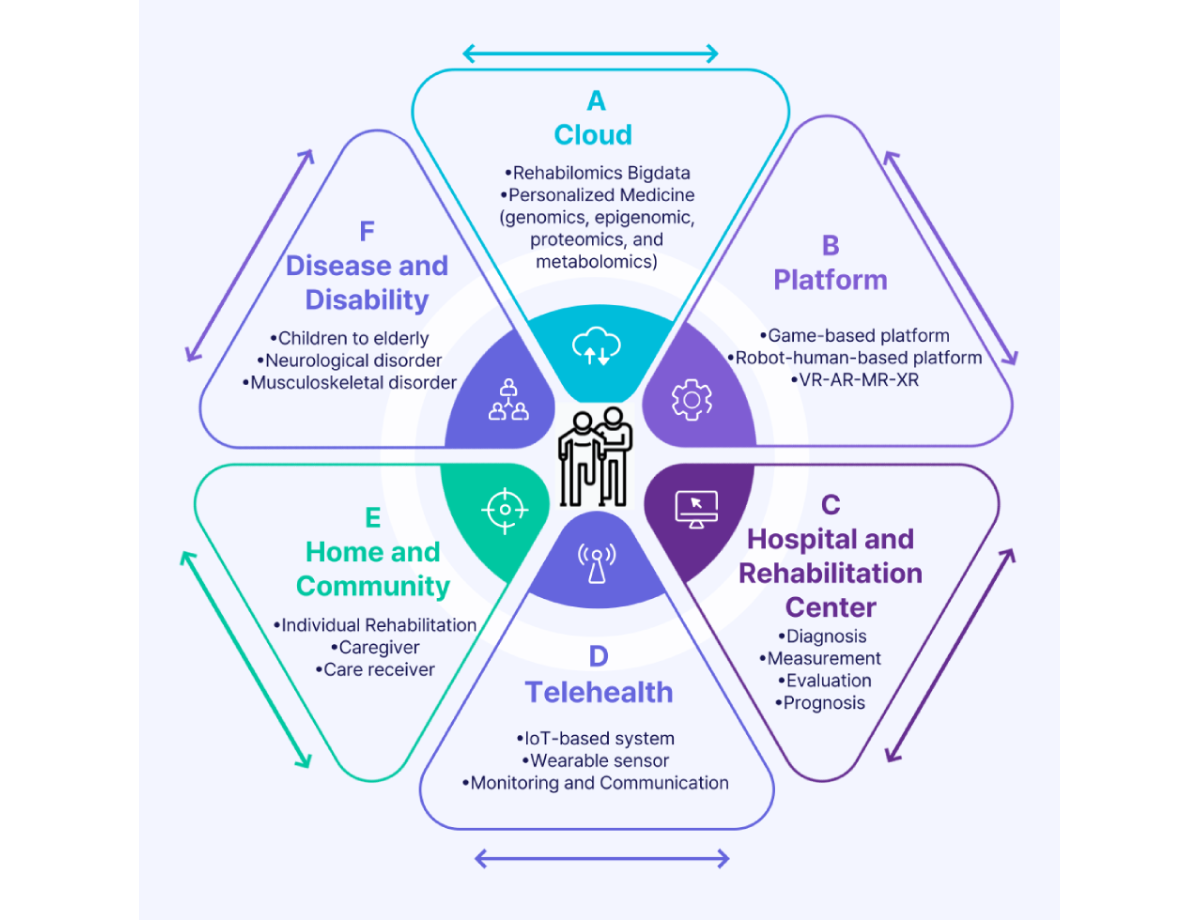
Rehabilomics strategies enabled by cloud-based rehabilitation. AR: augmented reality; MR: mixed reality; VR: virtual reality; XR: extended reality.

Our study established a strategy for personalized rehabilomics tailored toward individual needs by integrating clinical information from hospitals and extending it further to genomics, epigenomics, proteomics, and metabolomics for intervention, measurement, assessment, and prognosis (Figure 3). We emphasized the active use of clouds and platforms. In addition, we derived a strategic direction emphasizing the integration of hospitals and homes, promoting active community-based rehabilitation and linking it with individualized care providers. The first aspect involves a cloud that encompasses rehabilomics big data along with genomics, epigenomics, proteomics, metabolomics, and personalized medicine. The second aspect includes game-based platforms; robot–human-based platforms; and the use of VR, augmented reality, mixed reality, and extended reality. The third aspect involves hospitals and rehabilitation centers conducting diagnosis, measurement, evaluation, and prognosis, and using and linking such rehabilitation data. The fourth aspect is telehealth, which actively uses IoT-based systems, wearable sensors, and monitoring and communication. The fifth aspect involves home and community, using individual rehabilitation, and actively using information and rehabilitation between caregivers and care receivers. The sixth aspect focuses on disease and disability, actively using rehabilitation and data from children to the older adults, and targeting neurological and musculoskeletal disorders. All these processes, interconnected and used, ultimately lead to the development of a strategy for individualized rehabilitation.

## Discussion

### Principal Findings

#### Future Prospects of Cloud-Based Rehabilitation

The findings of this scoping review provide valuable insights into the prospects of cloud-based rehabilitation. Cloud computing technology offers innovative opportunities in the health care domain [44,54] and is increasingly being leveraged as part of rehabilomics strategies. This integrative approach has the potential to open new horizons in rehabilitation therapy and enhance patient-centered personalized treatment. Particularly, telehealth [55] and telemedicine rehabilitation [56,57] offer the advantage of providing medical support and monitoring through connectivity, especially for patients with chronic conditions, such as PD [16,17], stroke [42], and traumatic brain injury [11,12,58]. Daily routines and rehabilitation data are aggregated in the cloud, enabling individuals to review their personal data [49]. With their consent, patients can share these data with local community hospitals when seeking medical consultation or treatment, allowing health care professionals to assess their rehabilitation patterns at home [48].

Cloud-based rehabilitation related to the large data of rehabilomics and personalized medicine is needed [10,59]. This can be achieved through construction by a software company with cloud-related technology or using an existing cloud. Identifying aspects of cloud-based rehabilitation platforms to integratively manage patients’ exercise data, symptom reporting, and progress tracking will be helpful [60].

For example, cloud-based rehabilitation based on rehabilomics provides acute and subacute hospital treatment for patients with neurological damage, such as stroke and rehabilitation at home in the chronic phase [33,34,42,45,61,62].

Cloud-based rehabilitation based on rehabilomics continuously records cloud-based medical devices, rehabilitation exercises, and medications and visits the hospital during midterm evaluations to check records with rehabilitation experts [15,28,59]. This may help establish future intervention directions. In addition, combining web-based systems, such as telehealth, [63,64] with cloud-based rehabilitation, hospitals, and clinics allows intervention or evaluation to be performed in real time by linking with the medical system and is beneficial to both patients and caregivers [65,66]. Carrying out steady and sustainable rehabilitation is motivating, and it is cloud-based. A significant advantage exists in sharing and viewing various data in real time.

Hospitals can use these individualized data to collaborate, diagnose, and treat patients. Through the analysis of aggregated data among patient groups, they can offer research insights and develop tailored rehabilitation prescriptions. This facilitates the provision of personalized rehabilitation programs [48]. Particularly, it seems necessary to upgrade to an extensive and secure personal data system [18].

#### Security and Privacy Concerns

One of the prominent challenges associated with cloud-based rehabilitation is data security [67] and privacy. Ensuring confidentiality and integrity of patient data is of paramount importance and requires further research and policy development. Establishing stringent data security and privacy protocols between cloud service providers and health care institutions is essential, and patients must be confident that their data will be handled securely. Specifically, the protection of big data based on health care delivery and chronic disease management is necessary, particularly concerning systems integrating personal medical and rehabilitation information, such as application [68].

A cloud-based rehabilitation system needs to be integrated with electronic medical records and personal health records, based on individual consent for personal medical information and information protection policies founded on international standards, such as International Organization for Standardization and International Electrotechnical Commission 27018 for secure cloud services [69-71].

#### Enhancing Interdisciplinary Collaboration

The successful implementation of rehabilomics strategies underscores the importance of enhanced interdisciplinary collaboration [10,15]. Close cooperation between rehabilitation specialists, geneticists, data scientists, and health care information technology experts is expected to contribute significantly to the advancement of cloud-based rehabilitation and personalized therapy. Opportunities to foster dialogue and collaboration among researchers, clinical practitioners, and health care administrators should also be explored.

#### Future Research Directions

The consideration of future research directions in the field of cloud-based rehabilitation and rehabilomics strategies is crucial. Further research should prioritize the safety and efficiency of cloud services [67,72] based on blockchain technology [23,73-75], with a focus on data sharing and the development of standardized protocols [76,77]. More extensive clinical studies and evaluations are needed to validate the effectiveness of individualized rehabilitation programs and identify best practices for improving patient outcomes.

### Strengths of the Study

This study holds significant value for charting the development trajectory of cloud-based rehabilitation in the context of rehabilomics strategies. This study provides important insights by systematically analyzing the key factors for realizing personalized rehabilitation and emphasizing the importance of data security and interdisciplinary collaboration. Future studies should focus on developing standardized data-sharing protocols and rigorously evaluate the clinical efficacy of these approaches. Enhanced patient experience and treatment outcomes will serve as a driving force for advancing cloud-based rehabilitation. The findings of this study offer valuable information and directions for relevant researchers and clinical practitioners.

### Limitations

First, although the detailed variables and algorithms of each rehabilomics model were not known in the review process, various interventions and rehabilitation strategies according to each disease, body type, constitution, and gene are needed based on the models and strategies presented in this study. Second, this study did not involve a scoping review conducted by a librarian. However, the author received training on review search strategies from the affiliated library and proceeded with the research.

### Conclusions

This scoping review sheds light on the evolving landscape of cloud-based rehabilitation in the context of rehabilomics strategies. The integration of cloud computing technologies holds immense promise in revolutionizing rehabilitation practices and advancing personalized therapy. However, critical challenges must be addressed, particularly in the domains of data security and interdisciplinary collaboration.

Secure handling of patient data in cloud-based rehabilitation remains a paramount concern. As the field continues to expand, the development and implementation of robust data security and privacy measures are becoming increasingly nonnegotiable. This involves establishing stringent protocols, fostering transparency, and building trust among patients regarding data protection.

The success of rehabilomics strategies relies heavily on effective interdisciplinary collaboration. Collaboration between rehabilitation specialists, geneticists, data scientists, and health care IT experts should be encouraged. Bridging the knowledge gaps between these diverse fields is crucial for achieving comprehensive patient-centered care.

Future studies should prioritize the refinement of cloud services, emphasize data integrity, and develop standardized protocols for data sharing. Moreover, it is essential to conduct robust clinical studies to evaluate the efficacy of personalized rehabilitation interventions. These efforts will not only provide evidence of improved patient outcomes but also guide the implementation of best practices in clinical settings.

When harnessed effectively and responsibly, cloud-based rehabilitation has the potential to transform the field and usher in a new era of patient-centric data-driven therapy. As researchers and health care practitioners, it is our collective responsibility to navigate the challenges, collaborate across disciplines, and advance this promising frontier for better patient care.

## Appendix

Multimedia Appendix 1 PRISMA-ScR (Preferred Reporting Items for Systematic reviews and Meta-Analyses extension for Scoping Reviews) checklist.

Multimedia Appendix 2 Studies of cloud-based rehabilitation (N=28).

Multimedia Appendix 3 CONSORT-eHEALTH (Consolidated Standards of Reporting Trials of Electronic and Mobile Health Applications and Online Telehealth) checklist (V 1.6.1).

## Abbreviations

CoSHE: cloud-based smart home environment
IoT: Internet of Things
PD: Parkinson disease
PRISMA-ScR: Preferred Reporting Items for Systematic Reviews and Meta-Analyses Extension For Scoping Reviews
VR: virtual reality

## Data Availability

Data sharing is not applicable to this paper as no datasets were generated or analyzed during this study.
