# Supplementary material for: Rehabilomics Strategies Enabled by Cloud-Based Rehabilitation: Scoping Review
Source: J Med Internet Res. 2025 Jan 28;27:e54790. doi: 10.2196/54790 (PMC11815311; doi:10.2196/54790)
Supplement: Multimedia Appendix 2 [file jmir_v27i1e54790_app2.docx]

## .

**Table 1.** Study of cloud-based rehabilitation (N=28).

| Study, year, place of publication | Objective | Participants or technology | Cloud-based rehabilitation system | Main outcome and perceptions |
| --- | --- | --- | --- | --- |
| Fardoun et al [28], 2013, Saudi Arabia and Spain | A conceptual cloud-based framework with virtual and augmented reality support named “CRehab^a^” for the management of rehabilitation processes | A conceptual cloud-based framework with virtual and augmented reality support named “CRehab” | The rehabilitation wards, staff, patients, and families need systems that tackle administration, personal communication, and patient motivation processes effectively. To this end, CRehab uses cloud capabilities to address the first two concerns. Patient motivation is encouraged by the deployment of virtual rehabilitation environments with exercises that stimulate the patient. | To create feasible architecture for use in multiple types of rehabilitation, regardless of diagnosis and recovery processes, environment, patient, or any other factors (exercise program, services integration, etc). |
| Fortino and Gravina [29], 2014, Italy | BSNs^b^ are playing an important role in the ongoing revolution of the healthcare system, introducing the domain of mHealth^c^. | BSNs applications with cloud computing technologies | The integration of BSNs applications with cloud computing technologies is an emerging approach, promising to favor the diffusion of many mHealth services in real life. Among them, motor rehabilitation is one of the application areas where this is particularly true. Monitoring rehabilitation patients via communication networks and mobile computing systems is a crucial aspect because the idea of tying the opportunity to follow and monitor the patient at all postadmission stages through remote monitoring allows to substantially reduce the costs associated with the process. In contrast, patients that can safely perform rehabilitation and be monitored remotely will receive benefits in terms of comfort, less physical stress, and low economic cost. | A motor rehabilitation digital assistant, called Rehab-aaService, based on a 3-tier architecture that includes wearable motion sensor nodes, a personal mobile device, and a cloud-based backend supported by the BodyCloud middleware. |
| Hoda et al [30], 2014, Canada | To develop an intelligent, low-cost rehabilitation gaming system using a Microsoft Kinect sensor | A cloud-based rehabilitation exergame framework | They developed 2 goal-oriented exercises that target the rehabilitation of patients with chronic pain of the arm. These exercises include moving a ball into a net (basketball game) in the vertical plane and reaching a cup (reaching game) in the horizontal plane. In addition, accurate performance measures are provided for each exercise to quantitatively evaluate the effectiveness of the treatment plan for each patient. | The implemented system has increased user motivation to participate in the rehabilitation program, accelerated the recovery of the muscles, and offered the therapists the ability to assess the patients’ progress |
| Li and Pathirana [31], 2014, Australia | Exercise-based rehabilitation plays a vital role in the recovery of various conditions, such as stroke, PD^d^, and chronic pain. Recently, telerehabilitation has become increasingly popular. The quantitative nature in assessments, particularly for systematic monitoring of progress and cost saving for the patients as well as for the healthcare sector at large prove advantageous. However, challenges do exist in implementing a distributed biofeedback in a cost-effective and efficient manner. | Conceptual framework of a cloud-based telerehabilitation system using affordable noninvasive Microsoft Kinect. | The associated conceptual framework of a cloud-based telerehabilitation system using affordable noninvasive Microsoft Kinect allowing patients to perform rehabilitation exercises in nonclinical settings, such as home environments without losing quality of patient care. More importantly, different from existing telerehabilitation systems, our system not only measures whether patients can perform rehabilitation tasks, but also how well they can finish the tasks. | Preliminary experiments validate its potential in training healthy individuals to perform exercise motions emulating the physical rehabilitation process. |
| Škraba et al [32], 2015, Slovenia | The development of a prototype speech-controlled cloud-based wheelchair platform. The control of the platform is implemented using a low-cost WebKit speech API^e^ in the cloud. The description of the cloud-based wheelchair control system is provided. In addition to the voice control, a graphical user interface is implemented, which works in a web browser as well as on mobile devices providing live video streaming. | Speech-controlled, cloud-based wheelchair platform | Development was done in 2 phases: first, a small, initial prototype was developed and, second, a full-size prototype was built. The accuracy of the speech recognition system was estimated as ranging from approximately 60% to up to 97%, dependent on the speaker. The speech-controlled system latency was measured as well as the latency when the control is provided via touch on a smart device. Measured latencies ranged from 0.4 to 1.3 seconds. The platform was also clinically tested, providing promising results of cloud-based speech recognition for further implementation. | The developed platform is based on a Quad Core ARM Mini PC GK802 running Ubuntu Linux and an Arduino UNO microcontroller. Software development was done in JavaScript/ECMA^f^ Script, applying node.js |
| Pan et al [16], 2015, United States | This project sought to design, develop, and evaluate a prototype mobile cloud–based mHealth app, “PD Dr,” which collects quantitative and objective information about PD and would enable home-based assessment and monitoring of major PD symptoms. | Prototype mobile cloud–based mHealth app, “PD Dr” | They designed and developed a mobile app on the Android platform to collect PD-related motion data using the smartphone 3D accelerometer and send the data to a cloud service for storage, data processing, and PD symptom severity estimation. To evaluate this system, data from the system were collected from 40 patients with PD and compared with experts’ rating on standardized rating scales. | The evaluation showed that PD Dr could effectively capture important motion features that differentiate PD severity and identify critical symptoms. For hand resting tremor detection, the sensitivity was 0.77 and accuracy was 0.82. For gait difficulty detection, the sensitivity was 0.89 and accuracy was 0.81. In PD severity estimation, the captured motion features also demonstrated strong correlation with PD severity stage, hand resting tremor severity, and gait difficulty. The system is simple to use, user friendly, and economically affordable. The key contribution of this study was building a mobile PD assessment and monitoring system to extend current PD assessment based in the clinic setting to the home-based environment. The results of this study proved feasibility and a promising future for using mobile technology in PD management. |
| Hoda et al [33], 2015, Lebanon | They designed and implemented a cloud-based rehabilitation system that helps patients with stroke enhance their motor functions | The framework based on Kinect consists of 2 major components: the client component and the cloud component (45 healthy persons [18 females and 27 males] and 3 patients with stroke volunteered to participate in the experiment). | The ARIMA^f^ based on dynamic time warping algorithm for upper limb cloud rehabilitation and recovery prediction framework | The prototype of this system is tested on 3 patients for 10 weeks. Results show that patients have improved movement and control of their upper limbs over the course of training. This conclusion was also confirmed clinically; the patients performed the action research arm test under direct supervision of an orthopedic doctor and a professional physiotherapist. Moreover, recovery predictions with ARIMA models of patients with stroke have given encouraging results with a percentage of error <2.0% for patient 1 and patient 3, and 10.35% for patient 2. |
| Woodman et al [34], 2015, United Kingdom | Among the therapies available to patients with stroke, one that is gaining attention is the application of video games to encourage therapeutic movement. | Cloud-based therapy platform for upper limb rehabilitation and recovery after stroke. | The Limbs Alive project at Newcastle University has developed a system that gathers therapeutic game data from patients, uses statistical tools to estimate several performance metrics, and presents the results to patients and clinicians via web applications. | This paper describes the architecture of this system and outlines the various technical challenges that were overcome, including in security and deployment. |
| Khalifeh et al [35], 2016, Jordan | To design and implement an open-source eHealth monitoring and fall detection system to monitor senior patients. | Set of medical sensors and a microcontroller that communicates with the cloud. | The architecture of the system is based on a set of medical sensors and a microcontroller that communicates with the cloud. The sensors’ data are collected and processed using a microcontroller, the data are then stored on a cloud to form a permanent record, as well as to facilitate the diagnosis and the real-time monitoring for doctors, which plays a vital role in the patients’ rehabilitation and healing process. To fulfill this, a web interface was created that gave doctors the authority to access all patients’ medical records and monitor their health statuses. Furthermore, to detect whether the patient will experience unpredicted falls, a fall detection system is also considered. The system is composed of a microcontroller, voice recognition module, accelerometer, and a gyroscope. | Using the information gathered from the previous sensors, a fall can be detected and an alert message can be sent to the medical personnel for immediate help and assistance. |
| Causo et al [36], 2012, Singapore | A novel system was proposed that uses wearable wireless sensors and handheld devices for body motion tracking. The sensors are inertial measurement units and the handheld devices are smartphones and tablets. | Wireless wearable sensors and mobile  computing with cloud-based service | The system enables a patient who has experienced a stroke to perform rehabilitation exercises at home, eliminating frequent visits to the hospital. Furthermore, health care providers, such as therapists and doctors are able to keep track of their patient’s progress by remotely monitoring the rehabilitation activities of the latter. | Cloud services are integrated into system to allow remote monitoring, and replace expensive hardware and software licenses needed for data storage. Initial user tests show the feasibility of the system. |
| Mamun et al [17], 2017, Bangladesh | They proposed a cloud-based framework for detecting and monitoring patients with PD that will enable health care service in low resource settings. In developing countries, where most of the people do not receive proper health care services and are not well aware of PD, let alone detecting and receiving health care for PD, this system can be very practical and useful. | Telemonitoring applications to provide remote diagnosis of patients. | For this system, the patients of rural areas and patients from the regions where doctors are not available, can communicate to the doctors only if they have internet connections in their smart phones to access the cloud. Doctors can detect PD by checking the patient’s voice disorders or dysphonia over the cloud. With this system, a patient with PD can be detected easily and diagnosed by giving their voice samples through their phones, regardless of their location. | Based on the evaluation, the proposed systems are predicted to achieve 96.6% accuracy in the cloud environment for detecting PD. The proposed framework is expected to have great potential to enable health care services for patients with PD, who live in remote areas, especially in developing countries. |
| Bilic et al [37], 2017, Bosnia and Herzegovina, Turkey | An internet of things-based wearable system for physical rehabilitation monitoring and characterization. The system records movement data with 3-axial accelerometer and gyroscope sensors. | Internet of things-based wearable system for physical rehabilitation monitoring and characterization | Data recorded by the sensors are used for the characterization of movement, thus allowing for monitoring and estimation of the patients’ state at all times. The 3 main parts of the system are data acquisition unit, data-processing unit, and cloud-based service for remote access to data. Hardware implementation is described and shown for each of the 3 parts. The system is demonstrated for monitoring of elbow rehabilitation | The device can be used for highly precise and accurate monitoring of elbow flexion and extension characteristics, thus allowing for remote rehabilitation tracking through the use of the cloud-based service. |
| Cârstoiu et al [38], 2017, Romania | A cloud-based architecture for aphasia rehabilitation of patients having Romanian as their native language | Cloud-based architecture for aphasia rehabilitation | The platform comprises an application database, an application logic, and interfaces for patients and therapists. Accompanied by a virtual assistant, the patient has to solve exercises built by the therapist and, depending on the obtained scores, the treatment is adjusted, and data can be stored in a statistic module. | A scoring procedure is proposed, as part of the application logic and backup solutions for minimizing the estimated risks during the project’s life cycle are formulated. |
| Yee et al [39], 2017, Malaysia | A system-level design for an upper limb spasticity part-task trainer driven by clinical data stored in a cloud database | Cloud-based training device based on BITA 2.0 (methodology in developing the system-level design of the upgraded upper limb part-task trainer). | The robotic part-task trainer has been developed for preclinical training of medical personnel on the evaluation of upper limb spasticity based on the modified Ashworth scale. The cloud-based system enables continuous updating of clinical data of upper limb spasticity by rehabilitation physicians. Multiple part-task trainers can be connected to the cloud database via the internet, thus enabling the remote coaching of trainees and instant feedback of professional therapists and clinicians to the trainees. | It is expected that the functionality provided by the cloud technology will benefit the pedagogy of medical education in the future. |
| Inamura et al [50], 2021, Japan | Common sense and social interaction related to daily life environments are considerably important for autonomous robots, which support human activities. One of the practical approaches for acquiring such social interaction skills and semantic information as common sense in human activity is the application of recent machine learning techniques. Although recent machine learning techniques have been successful in realizing automatic manipulation and driving tasks, it is difficult to use these techniques in applications that require human-robot interaction experience. Humans have to perform several times over a long term to show embodied and social interaction behaviors to robots or learning systems. | Cloud-based VR^h^ platform research on human-robot interaction. | The cloud-based immersive VR platform that enables virtual human-robot interaction to collect the social and embodied knowledge of human activities in a variety of situations. To realize the flexible and reusable system, they developed a real-time bridging mechanism between ROS^i^ and Unity, which is one of the standard platforms for developing VR applications. The proposed system was taken to a robot competition field named RoboCup@Home to confirm the feasibility of the system in a realistic human-robot interaction scenario. Through demonstration experiments at the competition, it showed the usefulness and potential of the system for the development and evaluation of social intelligence through human-robot interaction. | The proposed VR platform enables robot systems to collect social experiences with several users in a short time. The platform also contributes in providing a data set of social behaviors, which would be a key aspect for intelligent service robots to acquire social interaction skills based on machine learning techniques. |
| Cheng et al [41], 2015, Taiwan | Somatosensory games for rehabilitation systems might be a trend. However, the cloud-based rehabilitation systems may be connected by the number of users. Therefore, performance and stability are very important. | Platform of the somatosensory game for cloud-based rehabilitation systems. | To find a suitable programming language to write this system, Java and Python were compared to test which one was better. A simulation and a monitoring system were designed to collect CPU^i^ and memory use. It was concluded that the Python server consumes fewer resources than the Java server. | The computing server is more effective in processing the data when Python is used in our environment. |
| Hossain et al [42], 2018, Saudi Arabia | To treat poststroke disability, cloud-based rehabilitation offers great advantages over conventional, clinic-based rehabilitation, providing ubiquitous flexible rehabilitation services and storage while offering therapeutic feedback from a therapist in real time during patients’ rehabilitative movements. With the development of sensory technologies, cloud computing technology integrated with AR^j^ may make therapeutic exercises more enjoyable. To achieve these objectives, this paper proposes a framework for cloud-based rehabilitation services, which uses AR technology along with other sensory technologies. | Cloud computing technology integrated with AR | They designed a prototype of the framework that uses the mechanism of sensor gloves to recognize gestures, to detect the real-time condition of a patient doing rehabilitative exercises. | There were statistically significant differences between the forces exerted by patients’ fingers at week 1 compared to week 6. Significant improvements in finger strength were found after 6 weeks of therapeutic rehabilitative exercises. |
| Borghese et al [43], 2018, Italy | Autonomous at-home rehabilitation through exergames requires clinicians to be able to quickly and reliably review patients’ progress and eventually tune exercise parameters for the next scheduled session. | Cloud-based platform featuring a flexible database, parametrized exergames, and a hierarchical description of the rehabilitation. | A cloud-based platform featuring a flexible database, parametrized exergames, and a hierarchical description of the rehabilitation data allows to achieve this goal. | A clear identification of different exercise macro parameters is fundamental to effectively display a clear and meaningful view of the patient’s status to the clinicians of the patient’s rehabilitation progress. |
| Pham et al [44], 2018, United States | The dramatic increase of senior populations worldwide is challenging the existing health care and support systems. Recently, smart home environments were used for ubiquitous health monitoring, allowing patients to stay in the comfort of their homes. | CoSHE^k^ for home health care | This paper presented a CoSHE for home health care. CoSHE collects physiological, motion, and audio signals through noninvasive wearable sensors and provides contextual information in terms of the resident’s daily activity and location in the home. This enables health care professionals to study daily activities, behavioral changes, and monitor rehabilitation and recovery processes. A smart home environment is set up with environmental sensors to provide contextual information. The sensor data are processed in a smart home gateway and sent to a private cloud, which provides real-time data access for remote caregivers. | It can successfully integrate contextual information into health data and this comprehensive information can help better understand caretakers’ health status. |
| Halloran et al [45], 2018, United Kingdom | A system enabling accurate remote assessment of stroke rehabilitation levels using wrist-worn accelerometer time series data. | Uses clustering models across sliding windows in the data and computation in the cloud. | The system is built based on features generated from clustering models across sliding windows in the data and makes use of computation in the cloud. | Predictive models are built using advanced machine learning techniques. |
| Jiang et al [46], 2018, China | ICT^l^ has shown its impact on medical research over the last few years. Big data analysis is adopted in many medical research applications, including rehabilitation after surgery. In the facial paralysis rehabilitation training progresses, traditional training processes require huge efforts from both patients and doctors. With increasing number of patients and limited resources offered by hospitals, assistance from ICT are urgently needed. | Cloud-based training and analysis system for patients with facial paralysis and physicians that provides rehabilitation training. The system provides automatic progress review and result evaluation. | A cloud-based training and analysis system for patients with facial paralysis and physicians that provide rehabilitation training. The system provides automatic progress review and result evaluation. A training client is developed to provide rehabilitation training as well as data collection. In addition, training results are analyzed by the cloud platform using machine learning methodologies. | The cloud platform provides the automatic evaluation of rehabilitation progress based on feedback from the training dataset and input from the physicians. |
| Sciuto et al [47], 2019, Italy | Psychological tests generally provide an evaluation scale to evaluate whether or not the individual manifests some traits. Such tests are generally used for attitude evaluation, personal selection, educational, and rehabilitation purposes, as well as for the diagnosis of cognitive disorders. | The main component for the administration of the cloud environment based on CAS^m^. | The use of tests and other question-based diagnostic tools represents one of the principal actions to start a clinical and therapeutic path, as well as for the evaluation and assessment of the possible educational and rehabilitation effort. Unfortunately, such tests are generally the result of a long and difficult process of validation for their standardization, simplification, and reorganizations driven by operations performed by means of complex statistical methods. In the work presented in this paper a unified cloud-based resource was developed for the management and execution of all the tasks related to psychometric testing, from the creation of a test to its validation and use. The solution has been designed to grant maximum flexibility allocating resources on a cloud service. | Moreover, by means of the distributed database, the solution is also able to support the simplification and reorganization process, as well as to serve as an online platform for the administration and consequent scoring of the finalized and standardized tests. |
| Wei et al [48], 2019, United States | The effectiveness of traditional physical therapy may be limited by the sparsity of time a patient can spend with the PT^n^ and the inherent difficulty of self-training given the paper/figure/video instructions provided to the patient with no way to monitor and ensure compliance with the instructions. | Cloud-based physical therapy monitoring and guidance system. | A cloud-based physical therapy monitoring and guidance system. It is able to record the actions of the PT as he/she demonstrates a task to the patient in an offline session and render the PT as an avatar. The patient can later train himself by following the PT avatar and receiving real-time guidance on his/her device. Because the PT and user (patient) motion sequences may be misaligned due to human reaction and network delays, a gesture-based dynamic time warping algorithm is proposed that can segment the user motion sequence into gestures, and align and evaluate the gesture subsequences, all in real time. They developed an evaluation model to quantify user performance based on different criteria provided by the PT for a task, trained with offline subjective test data consisting of user performance and PT scores. Moreover, three types of guidance were designed, which could be provided after each gesture based on user score, and subjective tests can be conducted to validate their effectiveness. | Experiments with multiple individuals show that the proposed system can effectively train patients, give accurate evaluation scores, and provide real-time guidance which helps the patients learn the tasks and reach satisfactory scores in less time. |
| Celesti et al [49], 2020, Italy | Nowadays, recent advancements in ICT have sped up the development of new services for smart cities in different application domains. One of these is definitely health care. In this context, remote patient monitoring and rehabilitation activities can take place either in satellite hospital centers or directly in citizens’ homes. Specifically, using a combination of cloud computing, IoT^o^ and big data analytics technologies, patients with motor disabilities can be remotely assisted avoiding stressful waiting times and overcoming geographic barriers. | TRaaS^p^ concept. | This paper focuses on the TRaaS concept. Such a service generates health care big data coming from remote rehabilitation devices used by patients that need to be processed in the hospital cloud. | Specifically, after a feasibility analysis, using a Lokomat dataset as a sample, we measured and compared the performances of four of the major NoSQL database management systems demonstrating that the document approach suits the case study. |
| Inamura et al [40], 2017, Japan | To determine the feasibility of the VR system—specifically, whether it has enough effect on SoA^q^ and SoO^r^ for healthy individuals—before conducting experiments for actual phantom limb patients. | A cloud-based VR platform for neurorehabilitation based on the SIGVerse system. | They developed a VR system that displays an avatar that has the same length as the subjective phantom limb. It determines the feasibility of the VR system—specifically, whether it has enough effect on SoA and SoO for healthy individuals—before conducting experiments for actual phantom limb patients. To this end, a VR system was developed in which a virtual avatar performs a motion identical to that of the participant by means of a motion-capturing device (Kinect V2). | The participant wears a 3D head mounted display (oculus Rift DK2) to experience seeing through the eyes of the avatar. Six conditions of avatar representation were used: 2 appearances of a normal human arm and a robot arm and three lengths of the arm (short, medium, and long). The participant executes elbow flexion-extension movement of the right arm, which causes the same movement in the VR avatar’s arm. After the induction movement, the subjective sense of the length of the right arm is measured by a pointing gesture of the left hand. Twelve individuals participated in this experiment. Results showed that the subjective length of the arm was changed according to the length of the displayed arm in the VR environment. From the results of a questionnaire, it was found that there is no negative effect on SoA. SoO when the participants watch the natural human avatar is stronger than when the robot arm is shown. These results are positive, thus confirming the basic potential of the proposed VR system. In conclusion, the change of self-body appearance of a VR avatar has enough effect on the subjective sense of arm length. Because the subjective sense of arm length is strongly related to body representation in the brain, the system can be a platform for research on embodied-brain science systems. |
| Yi et al [51], 2022, China | During the COVID-19 pandemic, the study considered the case of a child with DLD^s^ who could not go to the hospital on time to receive timely rehabilitation treatment due to disrupted hospital operations. The application of cloud-based rehabilitation platforms has provided significant advantages and convenience for children with DLD in-home remote rehabilitation. Among them, the JingYun Rehab Cloud Platform is the most widely used in mainland China. It is an interactive telerehabilitation system developed by Weixin Huang that delivers personalized home rehabilitation for special education children. | Remote cloud-based rehabilitation training exercises for children with DLD (n=162). | It used the JingYun Rehab Cloud Platform to investigate the extent to which cloud-based rehabilitation is effective for children with DLD in terms of language and cognitive outcomes. This was a prospective cohort study, including all children who were evaluated and diagnosed with DLD through sign-significant relations and were followed up at the rehabilitation clinic of the authors’ institute. The study followed 162 children with DLD for 3 months, including 84 children with DLD who participated in remote cloud-based rehabilitation on the JingYun Rehab Cloud Platform and 78 children with DLD were the control group who underwent home-based rehabilitation. Language abilities of both groups were assessed using the Chinese version of the PPVT-R. Several measures of training performance (language, memory, and cognition tasks) were assessed before and after cloud-based rehabilitation in the remote cloud-based rehabilitation group. Children with DLD in the cloud-based rehabilitation group performed significantly better in language abilities, as assessed by the PPVT-R, than children with DLD in the control group. Furthermore, for children who participated in remote cloud-based rehabilitation, the frequency of training sessions was proportional to their performance on language, memory, and cognition tasks. | This study demonstrated the effectiveness of cloud-based rehabilitation on the JingYun Rehab Cloud Platform in treating children with DLD with a significant improvement in the PPVT-R^t^ scores. |
| Wu et al [52], 2022, China | At both clinical and diagnostic levels, machine learning technologies could help facilitate medical decision-making. Prediction of sports injuries, for instance, is a key component of avoiding and minimizing injury in motion. Despite significant attempts to forecast sports injuries, the present method is limited by its inability to identify predictors. When designing measures for the avoidance of work-related accidents and the reduction of associated risks, the risk of injury to athletes is a crucial consideration. Various indicators are being evaluated to identify injury risk factors in several different methods. | The DLS^u^ for diagnosing sports injuries using the IoT and concept of cloud computing. | Consequently, this paper proposes a DLS for diagnosing sports injuries using the IoT and the concept of cloud computing. The IoT sensors that compose the body area network collect crucial data for the diagnosis of sports injuries, while cloud computing makes flexible computer system resources and computing power available. | This research examines the brain injury monitoring framework. It uses an optimal neural network to forecast brain injury and enhances the medical rehabilitation system for sports. Using the metrics accuracy, precision, recall, and *F*_1_-score, the performance of the proposed model is assessed and compared with current models. |
| Lv et al [53], 2023, Sweden | This work intends to enhance the standard of rehabilitative care provided to patients by optimizing the medical automation monitoring system enabled by wearable computing by edge cloud and IoT technology. First, the recent research literature on edge cloud and wearable computing devices was analyzed. Recent studies in virtual reality and automated medical rehabilitation were used to analyze and contrast various data fusion techniques in wearable sensors. Subsequently, an edge cloud model was constructed to enable real-time tracking of patients’ vital signs, allowing for timely assessment of their health status and rehabilitation progress. Then, a wearable device information monitoring rehabilitation system was established to provide effective rehabilitation treatment for patients with stroke. | Wearable computing by edge cloud and IoT technology. | The monitoring module of the rehabilitation system incorporates an edge computing terminal device, which models the virtual circuit for enhanced functionality. On the basis of the findings, when the number of output codes from the network structure is set to 1000, the average conversion energy of the dynamic conversion system is 140, the first conversion energy of the proposed model is 160, and the second conversion energy is 220. | Regarding the effectiveness of rehabilitation treatment, the system developed here demonstrates superior operational efficiency and delivers improved outcomes in rehabilitation treatment. This work serves as a practical reference for advancing the intelligent transformation of the medical service system. |

^a^CRehab: cloud rehab.

^b^BSN: body sensor network.

^c^mHealth: mobile health.

^d^PD: Parkinson disease.

^e^API: application programming interface.

^f^ECMA: European Computer Manufacturers Association.

^g^ARIMA: Auto Regressive Integrated Moving Average Model.

^h^VR: virtual reality.

^i^ROS: robot operating system

^j^CPU: central processing unit.

^k^AR: augmented reality.

^l^CoSHE: cloud-based smart home environment.

^m^ICT: information and communication technology.

^n^CAS: cloud administration service.

^o^PT: physical therapist.

^p^IoT: Internet of Things.

^q^TRaaS: telerehabilitation as a service.

^r^SoA: sense of agency.

^s^SoO: sense of ownership.

^t^DLD: developmental language disorder.

^u^PPVT-R: Peabody Picture Vocabulary Test-Revised.

^v^DLS: deep learning-assisted system.
